# Case Report: A Synonymous Mutation in *NF1* Located at the Non-canonical Splicing Site Leading to Exon 45 Skipping

**DOI:** 10.3389/fgene.2021.772958

**Published:** 2021-11-19

**Authors:** Pengzhen Jin, Kai Yan, Shaofen Ye, Yeqing Qian, Zaigui Wu, Miaomiao Wang, Yuqing Xu, Yanfei Xu, Minyue Dong

**Affiliations:** ^1^ Women’s Hospital, School of Medicine, Zhejiang University, Hangzhou, China; ^2^ Key Laboratory of Reproductive Genetics (Zhejiang University), Ministry of Education, Hangzhou, China; ^3^ Key Laboratory of Women’s Reproductive Health of Zhejiang Province, Hangzhou, China

**Keywords:** synonymous mutation, exon skipping, alternative splicing, whole-exome sequencing, NF1, neurofibromatosis type I

## Abstract

Synonymous mutations are generally considered non-pathogenic because it did not alter the amino acids of the encoded protein. Publications of the associations between synonymous mutations and abnormal splicing have increased recently, however, not much observations available described the synonymous mutations at the non-canonical splicing sites leading to abnormal splicing. In this pedigree, the proband was diagnosed Neurofibromatosis type I due to the presence of typical cafe’ au lait macules and pectus carinatum. Whole-exome sequencing identified a synonymous mutation c.6795C > T (p.N2265N) of the *NF1* gene which was located at the non-canonical splicing sites. Reverse transcription polymerase chain reaction followed by Sanger sequencing was carried out, and the skipping of exon 45 was observed. Therefore, the pathogenicity of the synonymous mutation c.6795C > T was confirmed. Our finding expanded the spectrum of pathogenic mutations in Neurofibromatosis type I and provided information for genetic counseling.

## Introduction

Neurofibromatosis type I (NF1, MIM 162200) is an autosomal dominant disease caused by mutations in the *NF1* gene (Stevenson and Viskochil, 2009), with the manifestations of cafe’ au lait macules, neurofibromas, Lisch nodules and bony dysplasia (Ferner et al., 2007; Sabbagh et al., 2009; Hernández-Imaz et al., 2013; Monroe et al., 2017). Over 3,000 mutations were recorded in the Human Gene Mutation Database (HGMD), among which, 583 lead to abnormal splicing.

It is a classic theory that the splice site mutations are causes for the abnormal splicing (Mort et al., 2014; Zhou et al., 2020). Missense mutations, nonsense mutations and frameshift mutations, with base substitutions and subsequently the changes of encoded amino acids, are the common variants (Jayasinghe et al., 2018). Numerous observations revealed that the synonymous mutations might cause abnormal splicing as well (Zhou et al., 2020; Knapp et al., 2021; Niersch et al., 2021). These mutations usually locate at the 3′ ends or 5’ ends of the exons, which are the canonical splicing sites (Mort et al., 2014; Jayasinghe et al., 2018; Kim et al., 2020). Recently, a few literatures described that synonymous mutations which located at the non-canonical splicing sites might cause abnormal splicing (Bechtel et al., 2008).

Herein, we report a pedigree of NF1. Whole exome sequencing (WES) identified the synonymous mutation c.6795C > T which led to the skipping of exon 45. This is the first report describing alternative splicing due to a synonymous mutation in the non-canonical splicing site of NF1, which expanded the mutation spectrum.

## Patients and Methods

### Case Report

The proband, a 3-year old female, was the first child of a healthy non-consanguineous couple. She was born at 38 weeks of gestation. Typical cafe’ au lait macules and pectus carinatum were appeared at 2 years old. Based on the manifestations, NF1 was diagnosed (Ferner et al., 2007; Monroe et al., 2017). The couple came for genetic counseling because the 28-year-old woman was at 15 weeks of gestation.

The current investigation was approved by the Ethics Committee of Women’s Hospital, School of Medicine Zhejiang University (IRB-20210259-R). All participants were provided their written informed consents.

### Genomic DNA and RNA Extractions

Blood samples were taken from the proband and her parents. Then, the QIAamp DNA Blood Kit (Qiagen, Germany) and RNeasy Midi Kit (Qiagen, Germany) were used to extract DNA and RNA, respectively, according to the manufacturer’s instructions. The concentrations of DNA and RNA were determined with using the NanoDrop 2000 (Thermo Fisher Scientific, United States).

### Whole-Exome Sequencing

DNA were fragmented and exomes were captured with the Agilent SureSelect Human All Exon Kit V5 (Agilent, United States). DNA was sequenced with 200bp reads by Illumina HiSeq2000 platform (Illumina, United States) as previously described (Zhu et al., 2019). Data were mapped to the Genome Reference Consortium Human genome build 37 (GRCh37) and only the variants locating in the coding sequence or splice site regions would be retained. Then, the candidate variants were filtered by frequencies on specific databases, including the Human Gene Mutation Database (HGMD), ClinVar database and genome Aggregation Database (gnomAD). The variants with allele frequency ≤1% would be retained. The Human Splicing Finder system (HSF, http://www.umd.be/HSF/) and the HOT-SKIP (http://hot-skip.img.cas.cz/) were used to predict the influence on the splicing.

### Reverse Transcription Polymerase Chain Reaction

Reverse transcription polymerase chain reaction (RT-PCR) was carried out to identify the splicing alternations. Then, the following primer sets (forward primer: 5′-ACG​TGC​AAG​TGG​CTG​GAC​CA-3’; reverse primer: 5′-GCA​GGT​GAA​GGA​TGC​CTG​TAC​CC-3′) were designed by primer5.0 to amplify the complementary DNA. (The detailed information about the primer sets were displayed in the supplement materials). The reaction was performed on the Thermal Cycler 9,700 (Applied Biosystems, Foster City, CA, United States). The procedure of the PCR was as following: denaturation at 94°C for 5 min, then 32 cycles at 94°C for 30 s, at 60°C for 30 s and at 72°C for 30 s, and a final extension at 72°C for 1 min. Then, the PCR products were analyzed by 3.0% agarose gel electrophoresis and sequenced as previously described (Jang et al., 2016).

## Results

### Identification of the Synonymous Mutation

**FIGURE 1:**
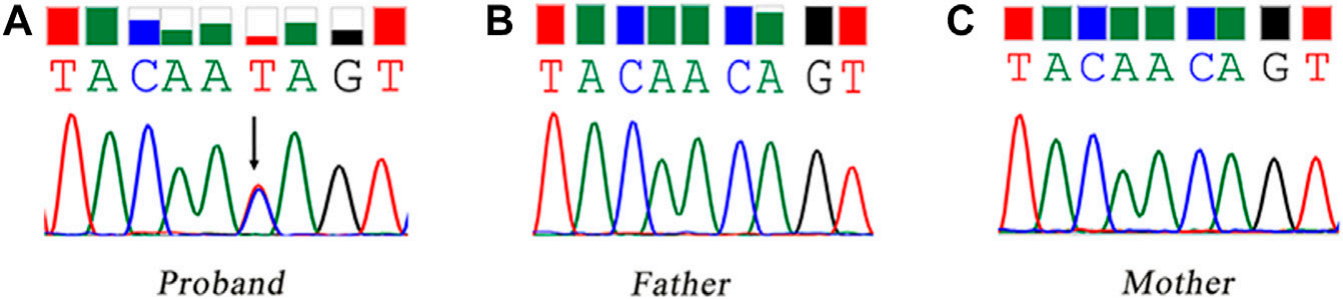
Sanger sequencing of the synonymous mutation of the *NF1* gene. The black arrow refers to the variant of c.6795C > T. **(A)** the proband, carrying the variant of c.6795C > T; **(B)** the father; **(C)** the mother.

WES identified a heterozygous mutation (NM_000267:c.6795C > T) in the *NF1* gene. It was a synonymous variant located at the middle of the exon 45 and had never been reported yet (PM2). The HOT-SKIP revealed that c.6795C > T increased the predicted ESS/ESE ratio from 0 to 1 (Table 1), which created an ESS or eliminated an ESE. To identify its parental origin, the variant was validated by Sanger sequencing and it was proved to be a *de novo* mutation (PS2) (Figure 1). According to the American College of Medical Genetics and Genomics (ACMG) and the Association for Molecular Pathology (AMP) guidelines (Richards et al., 2015), the mutation was predicted to be likely pathogenic (PS2*1 + PM2*1).

**TABLE 1:** Counts of Predicted ESSs and ESEs in the Wild-Type and c.6795C > T Alleles.

	Exon 45 segment	RESCUE-ESE	FAS-ESS	PESE	PESS	EIE	IIE	ESE total	ESS total	ESS/ESE
Wild type	TACAAC_6795_AGTCA	0	0	0	0	4	0	7	0	0
Mutant	TACAAT_6795_AGTCA	0	0	0	1	1	0	1	1	1

### Confirmation of the Exon 45 Skipping

**FIGURE 2:**
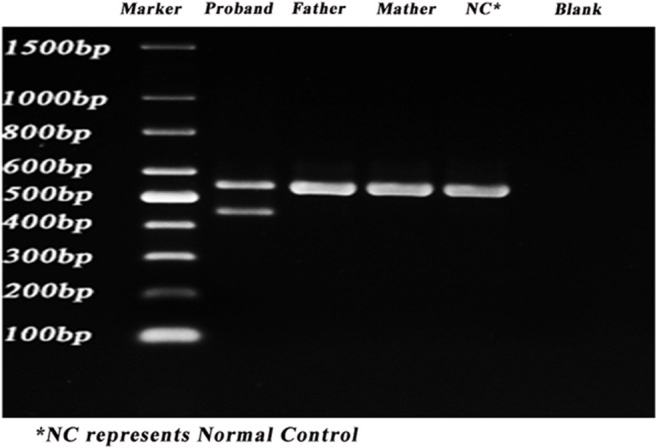
PCR results of the proband. PCR was carried out to identify the splicing alternations. Lane 1–6: marker, proband, father, mother, control, blank.

**FIGURE 3:**
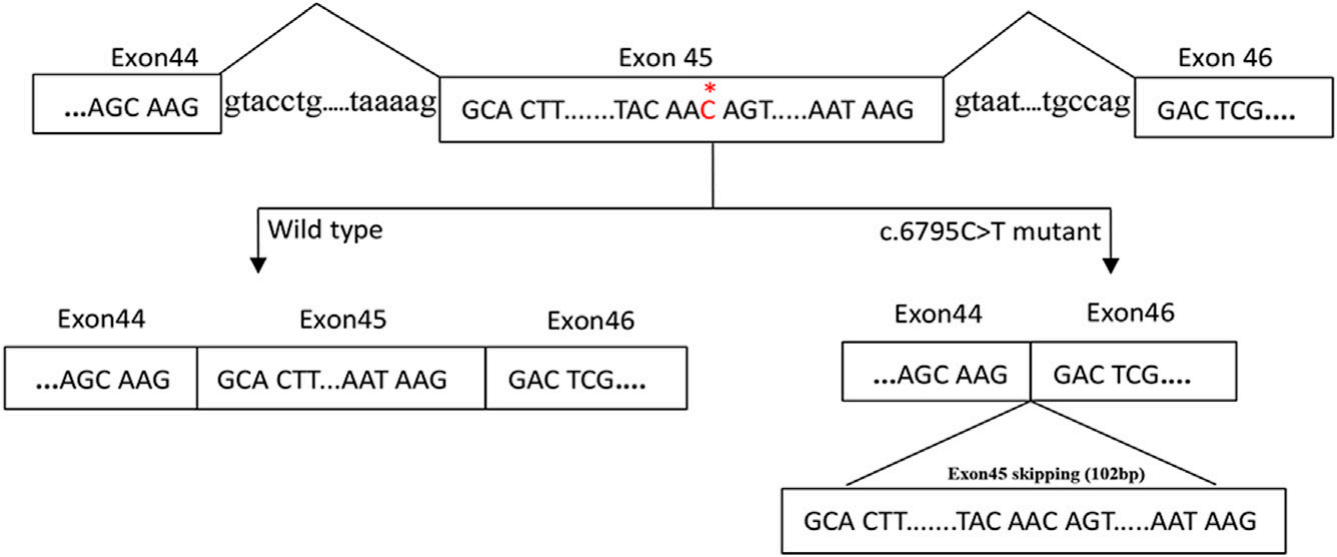
A proposed model for the role of the c.6795C > T mutation in exon skipping. The NF1 gene with part of the exons (box) and the flanking introns (out of box) is displayed in the first line. c.6795C > T mutation in exon 45 of NF1 gene induced the skipping of exon 45.

The gel electrophoresis indicated that (Figure 2), the bands had two bands of 548bp and 446bp in size, while the control and her parents had one band of 548bp. Subsequently, DNA isolated from the gel were sequenced and blasted. The band of the 548bp contained part of the 43, 44, 45, 46, 47, and part of exon 48, while the band of the 446bp contained part of the 43, 44, 46, 47, and part of exon 48. It was indicated that, the proband had the skipping of exon 45 (Figure 3), which led to the deletion of 34 amino acids.

To exclude the possibility that the mutations in the introns adjacent, primes were designed to amplify the introns 44 and 45. The Sanger sequencing did not revealed any variant in the introns 44 and 45 (data not shown).

## Discussion

In the current investigation, a *de novo* synonymous mutation c.6795C > T was identified in the proband who was diagnosed NF1, and this mutation was predicted to influence the splicing. RT-PCR and Sanger sequencing demonstrated the skipping of exon 45 with a deletion of 34 amino acids. This *de novo* synonymous mutation was scored to be likely pathogenic. This is the first report regarding a synonymous mutation of *NF1* gene influencing the alternative splicing (AS). Our findings expanded the mutation spectrum leading to NF1 and provided information for genetic counseling.

Compared with procaryotes, eukaryotes present a much higher degree of variety and complexity, which can be attributed in the function of AS (Richards et al., 2015). In higher eukaryotes, splicing discriminatively happens on different tissues or different stages of development, during the process of transcription, generating distinct transcripts from a single gene (Wang et al., 2008; Desmet et al., 2009). By selectively integrating or skipping specific exons in the pre-mRNA, AS serves as a means of genetic regulation to generate various mRNA, which encodes distinct proteins (Desmet et al., 2009; Fleming et al., 2012). Such mechanisms lead to an exponential increase in the number of the functional proteins and therefore, accounting for the biodiversity (Black, 2003; Pan et al., 2008; Desmet et al., 2009; Fleming et al., 2012; Mazin et al., 2021). According to the different changes occurred in the splicing sites, AS can be categorized into five types including exon skipping, alternative 5′ splice sites, alternative 3’ splice sites, mutually inclusive exons and intron retention (Black, 2003; Pros et al., 2006; Pan et al., 2008). To date, over 90% of human genes were observed to undergo the process of AS (Wang et al., 2008). The *NF1* gene, for example, is a typical gene reported to be enriched of the process of AS (Ars et al., 2000; Raponi et al., 2009).

Located on the chromosome 17q11.2, the *NF1* gene consists of 60 exons, some of which are alternatively spliced (Barron and Lou, 2012; Jang et al., 2016). Containing 2,818 amino acids, neurofibromin is the major protein encoded by the *NF1* gene, and expressed widely in the neurons, the Schwann cells and leukocytes (Zhu et al., 2002). It acts as a negative regulator to reduce the cell proliferation. Once the protein is functionally damaged, the cells will experience an unrestrained growth and finally leading to the neurofibromas, which are the typical clinical manifestations of the NF1 (Hutter et al., 1994; Monroe et al., 2017).

NF1 is one of the most common genetic disease with multi-system abnormalities involving the changes in the skin, muscle, neuronal system and other tissues derived from embryonic neuronal crest (Ferner et al., 2007; Sabbagh et al., 2009; Hernández-Imaz et al., 2013; Monroe et al., 2017). Generally, NF1 presents a high degree of variability because approximately 50% of mutations in the *NF1* gene were associated with splicing (Ars et al., 2000; Monroe et al., 2017). Frankly speaking, AS is an important mechanism leading to the biodiversity. Nevertheless, resulting from some unexpected mutations, specific AS may lead to pathogenic changes, likewise (Fleming et al., 2012). With an incidence of approximately 28% (Messiaen et al., 2000), the errors happened during the splicing process in *NF1* gene were estimated to be higher than other diseases (Raponi et al., 2009; Jang et al., 2016).

It is a classic theory that the canonical splice site mutations, located at the splice donor or the splice acceptor, mainly involving frameshift mutations, missense mutations and nonsense mutations, are the causes of the splicing sites disruptions (Mort et al., 2014; Jayasinghe et al., 2018; Kim et al., 2020). Synonymous mutations, also known as silent mutations (Liu et al., 2020), are defined as the base substitution that that don’t alter amino acids (Sauna and Kimchi-Sarfaty, 2011). Given the characteristics of the genetic code, synonymous mutations are usually considered non-pathogenic (Zhou et al., 2020). Nevertheless, studies proposed that the synonymous mutations could lead to abnormal splicing as well (Bampi et al., 2020; Horinouchi et al., 2020; Knapp et al., 2021; Niersch et al., 2021).

Actually, the classic theory above is not tenable when increasing number of evidences reveal that some mutations can result in the transcript alterations without damaging the canonical splice sites (Zhou et al., 2020). Different from the canonical splicing site mutations which located at the 3′ ends or 5’ ends of the exons, we identified the synonymous mutation c.6795C > T, which located at the middle of the exon 45 but lead to the exon 45 skipping. Previous studies have been reported that synonymous mutations can influence splicing by disrupting the splicing regulatory elements (SREs). SREs are specific nucleotide sequences that act as splicing regulators being targeted by SR proteins which is a protein that activates the splicing selections (Shin and Manley, 2002; Desmet et al., 2009). According to their functions, SREs are classified into the exonic splicing enhancers (ESEs), the exonic splicing silencers (ESSs), intronic splicing enhancers (ISEs) and intronic splicing silencers (ISSs) (Mort et al., 2014; Zhou et al., 2020). ESEs and ISEs help to integrate the exons or introns into the mRNA, while ESSs and ISSs conversely restrains the exons or introns from inclusion (Woolfe et al., 2010).

To our knowledge, it is the first report describing the exon 45 skipping caused by the synonymous mutation c.6795C > T which was located at the non-canonical splicing site. Our findings added a new mutation site into the spectrum of pathogenic mutations in *NF1* gene indicating that synonymous mutations, even at non-canonical splicing sites, should not be ignored.

## Data Availability

The datasets presented in this study can be found in online repositories. The names of the repository/repositories and accession number(s) can be found below: NCBI under BioProject number PRJNA776339 (https://www.ncbi.nlm.nih.gov/bioproject/PRJNA776339)
