# Supplementary material for: Case Report: A Synonymous Mutation in NF1 Located at the Non-canonical Splicing Site Leading to Exon 45 Skipping
Source: Front Genet. 2021 Nov 19;12:772958. doi: 10.3389/fgene.2021.772958 (PMC8640503; doi:10.3389/fgene.2021.772958)
Supplement: Supplementary file 1 [file Table1.DOCX]

Supplement material: The location of the primer sets

ACGTGCAAGTGGCTGGACCAGTGGACAGAACTAGCTCAAAGATTTGCATTCCAATATAATCCATCCCTGCAACCAAGAGCTCTTGTTGTCTTTGGGTGTATTAGCAAACGAGTGTCTCATGGGCAGATAAAGCAGATAATCCGTATTCTTAGCAAGGCACTTGAGAGTTGCTTAAAAGGACCTGACACTTACAACAGTCAAGTTCTGATAGAAGCTACAGTAATAGCACTAACCAAATTACAGCCACTTCTTAATAAGGACTCGCCTCTGCACAAAGCCCTCTTTTGGGTAGCTGTGGCTGTGCTGCAGCTTGATGAGGTCAACTTGTATTCAGCAGGTACCGCACTTCTTGAACAAAACCTGCATACTTTAGATAGTCTCCGTATATTCAATGACAAGAGTCCAGAGGAAGTATTTATGGCAATCCGGAATCCTCTGGAGTGGCACTGCAAGCAAATGGATCATTTTGTTGGACTCAATTTCAACTCTAACTTTAACTTTGCATTGGTTGGACACCTTTTAAAAGGGTACAGGCATCCTTCACCTGC

cDNA primer (Transcript : NM_000267.3)：

F：ACGTGCAAGTGGCTGGACCA

R: GCAGGTGAAGGATGCCTGTACCC

Table 1 The distribution of the products

| Location | The size of the products |
| --- | --- |
| Part of Exon43 | 41bp |
| Exon44 | 115bp |
| Exon45 | 102bp |
| Exon46 | 141bp |
| Exon47 | 127bp |
| Part of Exon48 | 22bp |
